# A prebreeding study of introgression spring bread wheat lines carrying combinations of stem rust resistance genes, Sr22+Sr25 and Sr35+Sr25

**DOI:** 10.18699/VJ21.081

**Published:** 2021-11

**Authors:** S.N. Sibikeev, O.A. Baranova, A.E. Druzhin

**Affiliations:** Federal Center of Agriculture Research of the South-East Region, Saratov, Russia; All-Russian Institute of Plant Protection, Pushkin, St. Petersburg, Russia; Federal Center of Agriculture Research of the South-East Region, Saratov, Russia

**Keywords:** bread wheat, introgressive lines, Sr22+Sr25 and Sr35+Sr25 gene combinations, prebreeding studies, мягкая пшеница, интрогрессивные линии, комбинации генов Sr22+Sr25 и Sr35+Sr25, пребридинговые исследования

## Abstract

The Sr22, Sr35, and Sr25 genes attract the attention of bread wheat breeders with their effectiveness against Puccinia graminis f. sp. tritici race Ug99 and its biotypes. The effectiveness and impact of Sr22+Sr25 and Sr35+Sr25 gene combinations on agronomic traits have not yet been studied. In the present article, these traits were studied using the spring bread wheat lines L503/W3534//L503, L503/Sr35//L503/3/L503 carrying the Sr22+Sr25 and Sr35+Sr25 genes during 2016–2020. These lines were assessed for resistance to P. graminis f. sp. tritici under natural epiphytotics and to the Saratov, Lysogorsk and Omsk populations of the pathogen and to the PgtZ1 (TKSTF) and PgtF18.6 fungus isolates in laboratory conditions (TKSTF + Sr33). The presence of the studied Sr-genes was conf irmed by using molecular markers. Prebreeding studies were conducted during 2018–2020 vegetation periods. Under the natural epiphytotics of the pathogen and in the laboratory conditions, the Sr22+Sr25 combination was highly effective, while Sr35+Sr25 was ineffective. For grain yield, the lines with the Sr22+Sr25 and Sr35+Sr25 genes were superior to the recipient cultivar L503 in one year (Sr22+Sr25 in 2019; Sr35+Sr25 in 2018), with a decrease in 2020, but in general there were no differences. For the period 2018–2020, both combinations showed a decrease in 1000 grains weight and an increase in the germination-earing period. The line with Sr22+Sr25 genes showed insignif icant effects on gluten and dough tenacity, but the ratio of dough tenacity to extensibility was higher, and f lour strength, porosity and bread volume were lower; in the line with Sr35+Sr25 genes, the gluten content was lower, but the strength, tenacity of the dough and the ratio of dough tenacity to extensibility were higher, f lour strength and the porosity of the bread were at the recipient level, but the volume of bread was lower.

## Abstract (ru)

Гены Sr22, Sr35 и Sr25 привлекают внимание селекционеров мягкой пшеницы эффективностью против расы Puccinia graminis f. sp. tritici Ug99 и ее биотипов. К настоящему времени защитный эффект комбинаций генов Sr22+Sr25 и Sr35+Sr25 не исследован, неизвестно их влияние на агрономические показатели. В представленной работе эти показатели изучены с использованием линий яровой мягкой пшеницы Л503/ W3534//Л503 (Sr22+Sr25) и Л503/Sr35//Л503/3/Л503 (Sr35+Sr25). Линии оценивали на устойчивость: к P. graminis f. sp. tritici в условиях естественных эпифитотий 2016–2020 гг., а также к саратовской, лысогорской и омской популяциям патогена и к изолятам гриба, PgtZ1 (TKSTF) и PgtF18.6 – в лабораторных условиях (TKSTF + Sr33). С помощью молекулярных маркеров подтверждено наличие изучаемых Sr-генов. Выявлена высокая эффективность комбинации генов Sr22+Sr25 как при естественных эпифитотиях патогена, так и в лабораторных исследованиях. Комбинация Sr35+Sr25 оказалась неэффективной. В среднем за 2018–2020 гг. у линий с обеими комбинациями генов отмечено понижение массы 1000 зерен и увеличение периода «всходы–колошение». У линии с комбинацией генов Sr22+Sr25 обнаружены незначительные эффекты на показатели клейковины и упругость теста, но отношение упругости теста к растяжимости было выше, а сила муки, пористость и объем хлеба – ниже; у линии с комбинацией Sr35+Sr25 количество клейковины ниже, но крепость, упругость теста и отношение упругости теста к растяжимости выше, сила муки и пористость хлеба на уровне реципиента, но объем хлеба ниже.

## Introduction

An increase in the harmfulness of the bread wheat diseases spectrum has been noted in the last decade for the zone of the Lower and Middle Volga regions of Russia. There was a strong epiphytoty of stem rust in 2016, a strong epiphytoty of leaf rust and Septoria tritici blotch (STB) in 2017, a strong epiphytoty of wheat tan spot in 2019 (Baranova et al., 2019; Sibikeev et al., 2020) and local but strong stem rust epiphytoty in 2020 (S.N. Sibikeev, unpublished data). Thus, there is a constant pressure of fungal diseases pathogens on bread wheat plants, in connection with which the increase in the yield of cultivated plants due to their resistance to biotic factors rises sharply.

Among the above-mentioned wheat diseases in the Saratov region, the role of the stem rust pathogen has sharply increased; the disease has become constantly present in the crops of spring and even winter bread wheat. So the development of Puccinia graminis f. sp. tritici on susceptible cultivars and lines of spring wheat was observed even in the dry growing seasons of 2018–2019. In terms of harmfulness, stem rust of wheat in the Saratov region began to occupy one of the first places. This fact is mainly explained by global climate changes in combination with agrobiological factors. Among the latter, two main factors are important. Firstly, most cultivars of bread wheat in the Saratov region are susceptible to this pathogen – namely, the following cultivars: Saratovskaya 55, Saratovskaya 68, Saratovskaya 70, Saratovskaya 73, Albidum 32, Favorit, Voevoda and Lebedushka. The cultivars Prokhorovka, Yugo-Vostochnaya 2 and Dobrynya were heterogeneous in terms of resistance (Baranova et al., 2019), the second factor is the presence of highly virulent population of P. graminis f. sp. tritici. So in the Saratov populations for 2016–2020 the proportion of highly virulent pathotypes (from 14 to 20 virulence genes) ranged from 35 to 60 % (O.A. Baranova, unpublished data).

To avoid economically significant losses from bread wheat diseases, including stem rust, constant scientifically based breeding for resistance to pathogens is required. This work should be based on knowledge of the pathogen biology, its virulence, resistance genetics of cultivated varieties and on a sufficient number and diversity of genes for resistance to pathogens; that is, it should be anticipatory (McIntosh, 1992; McIntosh, Brown, 1997).

As breeding practice shows, the most difficult task to solve is the expansion of the genetic diversity of effective resistance genes. Thus, studies by O.A. Baranova (2020) on a set of 32 new bread wheat cultivars, included in the “State Register of Breeding Achievements” of the Russian Federation for 2017–2018, showed that only 11 cultivars are highly effective against the causative agent of stem rust. Seven of them are protected by one Sr31 gene, three cultivars – by the combination of Sr31+ Sr57 genes, and one cultivar – by the combination of Sr31+ Sr28 genes. Thus, protection can be determined by only one Sr31 gene, since the interaction of genes in combinations has not been proven. In addition, the situation is complicated by the fact that the Sr31 gene has been overcome by the Ug99 race, which currently consists of 13 biotypes (http;/globalrust.org/pathogens/pathogen-homepage). The Ug99 race is widespread in the countries of Africa and the Middle East, it migrates in the direction of Central and Southeast Asia, and it is possible to introduce it into the territory of the Russian Federation. In this regard, it is necessary to take this fact into account and include in the breeding process of bread wheat cultivars resistant to P. graminis f. sp. tritici the Sr-genes and their combinations effective against the biotypes of the Ug99 race.

At present, of the total number of identified genes for resistance to stem rust of wheat, 29 out of 61 have been transferred from the “alien” species (McIntosh et al., 2013, 2016, 2018, 2020). The genes Sr25, Sr22, and Sr35 occupy a special place among them. They are all effective against the Ug99 race and its biotypes (http://rusttracker.cimmyt.org/?page_id=22). The Sr25 gene was transferred from the tall wheatgrass Agropyron elongatum (2n = 70) into bread wheat as part of the 7DS- 7DL-7Ae#1L translocation, the last two from the A genome of the cultivated einkorn into chromosomes 7AL and 3AL, respectively (McIntosh et al., 1995).

While the Sr25/Lr19 gene complex (a gene for resistance to leaf rust) is widely used in cultivars and breeding material of spring bread wheat in the Middle Volga and Lower Volga regions (Gultyaeva et al., 2019, 2020), the Sr22 gene is used only in the cultivars Schomburgk and BT-Schomburgk in Australia and in a set of near isogenic lines, and the Sr35 gene has not been introduced into commercial cultivars (McIntosh et al., 1995, 2013). The limited use of the Sr22 and Sr35 genes in practical breeding is mainly due to the fact that they either do not compensate for the absence of wheat chromatin or contain undesirable genetic factors with negative effects (Paul et al., 1994). The Sr25/Lr19 genes, or rather the 7DS- 7DL-7Ae#1L translocation, has a positive effect on agronomic traits (Singh et al., 1998; Sibikeev et al., 2016, 2018). It was noted that an increase in grain productivity in the presence of this translocation is determined by a better utilization of assimilates by the reproductive organs (Miralles et al., 2007). However, to date, studies of the Sr22+Sr25 and Sr25+Sr35 gene combinations both in terms of effectiveness against the stem rust pathogen and the effect of introgressed genetic material on agronomically important traits (prebreeding studies) have not been carried out.

The aim of our research was to reveal the promising nature of the Sr22+Sr25 and Sr35+Sr25 gene combinations for practical breeding both in terms of effectiveness against P. graminis and in terms of their effect on productivity and grain quality.

## Materials and methods

The material used included the following genotypes of spring bread wheat: cultivar – recipient L503, contains the 7DS-7DL- 7Ae#1L translocation with the Sr25/Lr19 genes (Badaeva et al., 2018); standard for the Saratov region cultivar Favorit, contains the substitution 6D(6Agi) (Sibikeev et al., 2017).

Introgression lines: L503/W3534//L503, where W3534 is a near isogenic line of the Marquis cultivar with the Sr22 gene, namely W3534 = Marquis*5//Stewart*3/T. monococcum; L503/Sr35//L503/3/L503, where Sr35 is a near isogenic line of the Marquis cultivar with the Sr35 gene, namely Sr35 = Marquis*5//G2919, G2919 Canadian source of T. monococcum. The lines W3534 and Sr35 were kindly provided by Dr. R.A. McIntosh (Plant Breeding Institute, Gobbitty, Australia) and were used as paternal forms for crossing with spring bread wheat cultivar L503.

The studies included three stages: the first stage was to confirm the presence of the Sr22+Sr25 and Sr35+Sr25 combinations in the studied introgression lines. Sr-genes were identified using molecular markers for Sr25 (Gb) (Prins et al., 2001), Sr22 (Xbarc121, Xcfa2123, Xcfa2019, Xwmc633) (Khan et al., 2005; Yu et al., 2010), Sr35 (Xcfa2170) (Zhang et al., 2010). Amplification was performed on C1000 Thermal Cycler (BioRad) amplifiers; amplification products were separated in 2 % agarose and 8 % polyacrylamide gels stained with ethidium bromide. The SWSR22TB line containing the Sr22 gene and the W3435 (Sr22) parental line, as well as the Marquis*5//G2919 (Sr35), LC-SR25-ARS (Sr25) line was used as a positive control. The susceptible cultivar Khakasskaya served as a negative control, and a PCR mixture without the addition of DNA served as a control for contamination. A GeneRuler™ 50 bp DNA Ladder (“Fermentas”) was used as a molecular weight marker. The amplification products were visualized using the ChemiDoc XRS+ (BioRad) gel documenting system. PCR was performed in two replicates.

The second stage was an evaluation of the lines resistance to the causative agent of stem rust in the field conditions in 2016–2020 – the phase of milky-wax ripeness (breeding sowing by the Federal Center of Agricultural Research of the South-East Region) against the natural background of the pathogen development. The stem rust infection type was determined using the A.P. Roelfs et al. (1992) scale, where R is resistant, MR is moderate resistant, MS is moderate susceptible, and S is susceptible, respectively. The degree of rust damage (%) was assessed according to the scale of R.F. Peterson et al. (1948). In the phase of seedlings (first leaf ) in All-Russian Research Institute of Plant Protection, juvenile resistance of wheat samples to disease was studied according to the method of Y. Jin et al. (2007). Ten-day-old seedlings with a fully unfolded first leaf were inoculated with a urediniospore suspension of pathogen populations collected in the Omsk region, as well as in the Lysogorsky district of the Saratov region from the Favorit cultivar, which carries the 6Agi(6D) substitution, as well as two isolates of the fungus – PgtZ1 (TKSTF) and PgtF18.6 (TKSTF + Sr33). The virulence characteristic of the PgtZ1 and PgtF18.6 isolates is shown in Table 1.

**Table 1.**
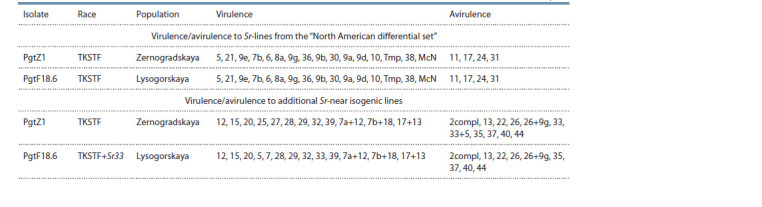
Virulence characteristic of P. graminis f. sp. tritici isolates used to inoculate introgression wheat lines in the seedling stage

The inoculums concentration was 1 mg of urediniospores in 1 ml water (Singh et al., 2008). The Khakasskaya spring bread wheat cultivar was used as a susceptible control. The results were taken into account on the 10th day according to E.C. Stakman et al. scale (1962), where 0 is the absence of symptoms; 0; – necrosis without pustules; 1 – very small pustules surrounded by necrosis; 2 – pustules of medium size, surrounded by necrosis or chlorosis; 3 – pustules of medium size without necrosis, 4 – large pustules without necrosis, X – pustules on the same leaf of different types, chlorosis and necrosis are present. Plants with reaction types 0, 0; 1, 2 were considered resistant, and 3, 4 and X were considered susceptible.

The third stage is the evaluation of grain productivity traits, physical properties of the dough and baking indicators in the introgression lines L503/W3534//L503 (Sr22+Sr25) and L503/Sr35//L503/3/L503 (Sr35+Sr25) in comparison with the recipient cultivar L503 and the standard cultivar Favorit. The studies were carried out in 2018–2020, of which 2020 was the most favorable; however, during this growing season, there was a deficit of precipitation from the flowering phase to full ripeness, and 2018 and 2019 were distinguished as severely droughty throughout the entire field season.

The experimental material was randomly sown in 7 m2 plots in three replicates. The seeding rate was 400 grains per 1 m2. The bread making quality was evaluated by the content of crude gluten, gluten strength and the indicators of the IDG-1 device (deformation index of gluten) and the Chopin alveograph with the baking of experimental bread samples. The protein content of grain, harvested in 2020, was determined on the Infratec™ 1241 Grain Analyzer. The data obtained were subjected to the appropriate statistical analysis using the Agros-2.10 software.

## Results

Identification of resistance genes

To confirm the presence of Sr22+Sr25 and Sr35+Sr25 gene combinations in the introgression lines L503/W3534//L503 and L503/Sr35//L503/3/L503, Sr-genes were identified using molecular markers of the Sr-genes under study.

**Fig. 1.**
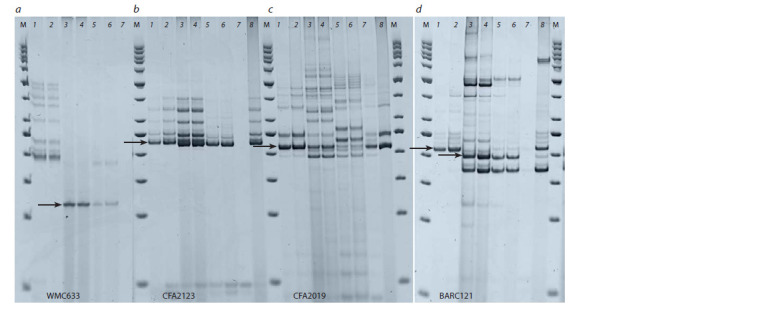
Identif ication of the Sr22 gene using molecular markers Хwmc633 (a), Хcfa2123 (b ), Хcfa2019 (c) and Xbarc121(d). M – marker of molecular weight 50 bp “Fermentas”; No. 1, 2 – line L503/W3534//L503; positive control of Sr22: 3, 4 – line W3534, 5, 6 – line SWSR22TB; negative control of Sr22: 7 – cultivar Khakasskaya, 8 – cultivar Inna.

The Sr22 gene is introgressed into tetraploid wheat from Triticum monococcum L. ssp. aegilopoides (synonym T. boeoticum Boiss.). For its identification, three molecular markers closely linked to it are usually used – Xcfa2019, Xcfa2123 and Xbarc121 (Yu et al., 2010). In the work (Olson et al., 2010), a set of lines with Sr22 gene was obtained and the nearest flanking markers of this gene, Xwmc633 and Xcfa2123, were proposed. In our work, we used all four Sr22 markers: Xbarc121, Xcfa2123, Xcfa2019 and Xwmc633 (Fig. 1).

The size of the obtained PCR products with markers Xbarc121, Xcfa2123, Xcfa2019 and Xwmc633 is shown in Table 2. It was shown that when PCR was performed with the primers barc121F/R, cfa2123F/R, cfa2019F/R and wmc633F/R, fragments of different sizes were amplified and not only those that were declared as diagnostic. Thus, during amplification with primers wmc633F/R in the lines SWSR22TB and W3534 a diagnostic fragment of 117 bp size was obtained. In the introgression line L503/W3534// L503, the obtained fragment was about 211 bp. In the work of E.L. Olson et al. (2010), in the line U5616-20-154 with the small fragment of T. monococcum during amplification with primers wmc633F/R, a fragment of 229 bp was obtained, which was explained by recombination between the resistance gene and all markers mapped in this area.

**Table 2.**
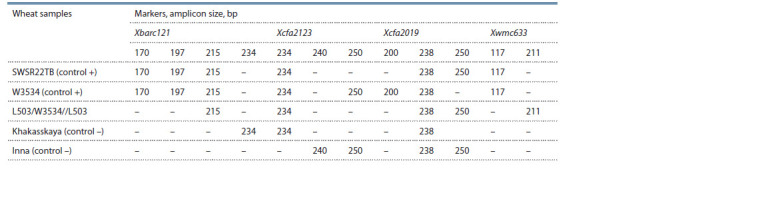
Polymorphism by the size of amplification fragments of molecular markers of the stem rust resistance gene Sr22 Notе. “–” – no amplicon.

For the Xbarc121 marker, the 215 bp size amplicon described as a diagnostic fragment by L.X. Yu et al. (2010) was observed in the control lines SWSR22TB and W3534, as well as in the line L503/W3534//L503. When analyzing the PCR products for the Xcfa2123 marker, our results coincided with the data of J.K. Haile et al. (2013). In the control lines SWSR22TB and W3534, the fragment of 234 bp size was amplified. A similar fragment was observed in the L503/W3534// L503 line, but it was also observed in the Khakasskaya cultivar. The amplicons size of the Inna cultivar was somewhat larger – 240 and 250 bp. Amplification with cfa2019F/R primers in the SWSR22TB line revealed two fragments – 238 and 250 bp in our work, as in the line L503/W3534//L503. The line W3534 had fragments of 200 and 238 bp. However, it should be noted that the 238 bp fragment was also amplified in negative controls – the cultivars Khakasskaya and Inna. Amplification of the 238 bp diagnostic fragment for Xcfa2019 was also shown in the work of E.L. Olson et al. (2010), which does not coincide with the data of J.K. Haile et al. (2013).

Thus, amplicons were identified by three markers to Sr22 (Xbarc121, Xcfa2123, Xcfa2019) in the line L503/W3534// L503. In addition, this line was resistant to P. garminis isolates PgtZ1 and PgtF18.6, avirulent to the line with Sr22 and virulent to the line with Sr25, and based on the pedigree data and identification of resistance genes, there are no other Sr- genes in this line. On this basis, we concluded that the L503/W3534// L503 line contains Sr22 gene.

**Fig. 2.**
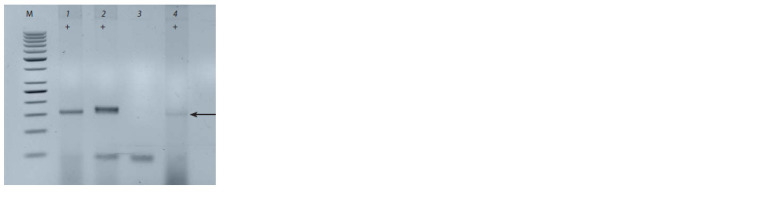
Identif ication of the Sr35 gene using the molecular marker Xcfa2170. M – marker of molecular weight 50 bp “Fermentas”; No. 1 – line L503/Sr35// L503/3/L503; positive control of Sr35: 2 and 4 – line Marquis*5/G2919 (Sr35); negative control of Sr35: 3 – cultivar Khakasskaya. The arrow indicates diagnostic fragment with molecular weight of 160 bp

The Sr35 gene was identified using the Xcfa2170 marker (Fig. 2) in the L503/Sr35//L503/3/L503 line. The parental line Marquis*5/G2919 (Sr35) was used as a control.

When the Sr35 gene was identified using the Xcfa2170 marker, a 160 bp diagnostic fragment was obtained in positive controls, which coincides with the data of J.K. Haile et al. (2013). In addition to the Sr22 and Sr35 genes, Sr25/Lr19 was also identified in the L503/W3534//L503 and L503/ Sr35//L503/3/L503 lines using the Gb marker. It should also be noted that the L503/Sr35//L503/3/L503 line was resistant to the Ug99 race in Kenya (Baranova et al., 2021).

Thus, it was proved that the introgression lines L503/ W3534//L503 and L503/Sr35//L503/3/L503 carry the combinations of Sr22+Sr25 and Sr35+Sr25 genes, therefore, the results of phytopathological and prebreeding studies presented below are correct.

Phytopathological analysis of resistance to the stem rust causative agent

Analysis of the reaction type to the stem rust causative agent was carried out both in the field with natural epiphytotiсs of the disease, and in laboratory conditions with artificial infection of seedlings. Evaluation of resistance to P. graminis f. sp. tritici under the conditions of epiphytotics 2016–2020 showed that the line L503/W3534//L503 (Sr22+Sr25) showed the type of reaction to the pathogen R, while in the line L503/ Sr35//L503/3/L503 (Sr35+Sr25)showed the type of reaction R was at epiphytotics of 2016–2019, and in 2020 – 20MS. At the same time, the reaction type of the recipient cultivar L503 (Sr25) in 2016, 2017 and 2020 was 25MS, 20MS, 30MS and in 2018, 2019 – R, and the reaction type to the pathogen of the cultivar Favorit (6D(6Agi) LrAgi/SrAgi) in epiphytotics of P. graminis f. sp. tritici in 2016–2020 was 75S, 50S, 10S, with the exception of 2019, when the 5S and R reaction types were observed on plants. This was caused by a severe drought and poor development of P. graminis f. sp. tritici in 2019 (Table 3).

**Table 3.**
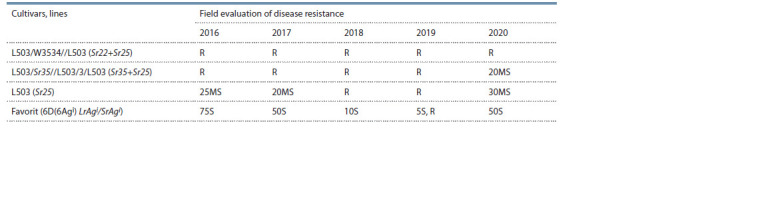
Field evaluation of spring bread wheat introgression lines for resistance to P. graminis f. sp. tritici in 2016–2020 (stage “milky-waxy ripeness”)

The results of the evaluation of resistance to the stem rust in laboratory conditions at the seedlings stage are shown in Table 4. Laboratory evaluation at the seedlings stages of the studied lines showed resistant responses to the pathogen in the line with the Sr22+Sr25 gene combination (ITs ranging from 0 to 2) and susceptible in the line with the Sr35+Sr25 gene combination. It should be noted that these reaction types were related to both P. graminis f. sp. tritici populations and PgtZ1 and PgtF18.6 isolates.

**Table 4.**
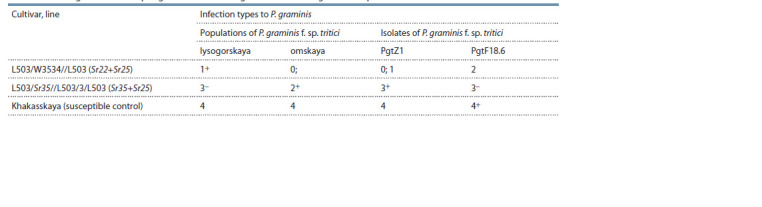
Seedling resistance of spring bread wheat introgression lines to P. graminis f. sp. tritici

Thus, the phytopathological analysis of resistance to the stem rust causative agent of introgression lines L503/W3534// L503 (Sr22+Sr25) and L503/Sr35//L503/3/L503 (Sr35+Sr25), both in natural epiphytotics of P. graminis and with artificial infection, showed high and effective resistance of the Sr22+Sr25 combination and the susceptibility of the Sr35+Sr25 combination during epiphytotics in 2020 and laboratory evaluation.

Prebreeding studies of introgressive lines

The results of studying grain productivity in the introgression lines L503/W3534//L503 (Sr22+Sr25) and L503/Sr35// L503/3/L503 (Sr35+Sr25) showed that, on average, for the period from 2018 to 2020, there were no significant differences in lines for grain yield compared to the recipient cultivar L503 and the standard cultivar Favorit (Table 5), which was expected, since the productivity indicators in 2020 were twice as high as the grain yield in 2018 and 2019. Nevertheless, the analysis of grain productivity by years revealed that, in 2018, under the background of severe drought throughout the growing season, the yield was the highest in the line with the Sr35+Sr25 combination (significant excess of the recipient cultivar L503 and at the level of the standard cultivar Favorit). In the same line, there was an insignificant excess in grain yield of the cultivar L503 in the growing season of 2019 with a similar drought as in 2018. However, the line with the Sr35+Sr25 combination was significantly lower for grain productivity under the conditions of the 2020 growing season, which was characterized by excess moisture and moderate air temperature from germination to the beginning of flowering, then a drought with high temperatures was noted until full maturation.

**Table 5.**
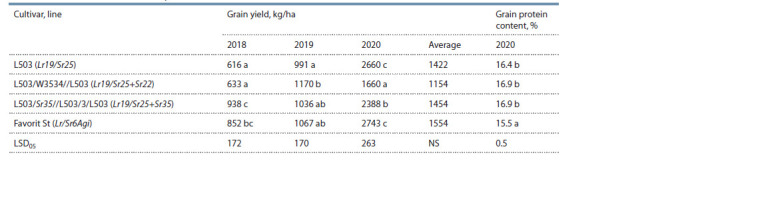
The grain productivity of spring bread wheat introgression lines with the Sr22+Sr25 and Sr35+Sr25 genes combination and cultivars L503 and Favorit for the period of 2018–2020

In general, for three years of testing in terms of the absolute indicator of grain yield, L503/Sr35//L503/3/L503 (Sr35+Sr25) is equal to the recipient cultivar L503. L503/ W3534//L503 (Sr22+Sr25) for grain yield was at the level of L503 in 2018, exceeded L503 in 2019 and was significantly lower than the cultivars L503 and Favorit in 2020. In general, over three years, in terms of absolute numbers for grain productivity, the line L503/W3534//L503 (Sr22+Sr25) is inferior to both L503 and Favorit. The line L503/Sr35//L503/3/L503 (Sr35+Sr25) is a more productive line (in absolute numbers), which was revealed when comparing the studied lines with each other.

On average, for 2018–2020, the analysis of 1000 grains weight, as one of the important elements of grain productivity, showed a significant decrease in lines with the combination of Sr22+Sr25 (28.2 g) and Sr35+Sr25 (30.1 g) genes compared with the recipient cultivar L503 (31.3 g). Moreover, this decrease was larger for the line with Sr22+Sr25, which was significantly inferior to Sr35+Sr25, while for the standard cultivar Favorit – 28.0 g, with LSD05 = 0.95 g and F* = 9.67. On average, for 2018–2020, in terms of the germination to earing period, significant differences were observed between the recipient cultivar L503 (44.3 days) and lines with Sr22+Sr25 (47.7 days) and Sr35+Sr25 (46.7 days) gene combinations, the differences between the lines were not significant, with LSD05 = 1.0 days and F* = 27.60. There were no differences in plant height between the studied lines and the cultivars L503 and Favorit.

An important stage in the production of bread wheat cultivars is the quality of the final product – flour and bread.

Unfortunately, it is not uncommon for the involvement of alien genetic variability in the bread wheat gene pool to worsen some indicators of flour and bread quality. Over the period of research, it was revealed that the lines with the combinations Sr22+Sr25 and Sr35+Sr25 and the recipient cultivar L503 did not have significant differences in protein content, but exceeded the standard cultivar Favorit (see Table 5) on average. According to the indicators of gluten – the content and strength of gluten according to the IDG-1 device indicators, the following results were obtained: the line with the Sr35+Sr25 combination significantly reduced the gluten content, but strength was significantly higher in relation to the cultivar L503, and the line with Sr22+Sr25 combination did not differ from the recipient cultivar L503 according to this indicators. The values of dough tenacity indicators and the ratio of dough tenacity to extensibility (P/L) were distributed as follows: higher for the combination Sr35+Sr25, and the combination Sr22+Sr25 did not differ in tenacity from the cultivar L503, but had a higher tenacity to extensibility ratio (P/L). The line with the Sr22+Sr25 combination significantly reduced the flour strength, the crumb porosity and bread volume in relation to the recipient cultivar L503. At the same time, the line with the Sr35+Sr25 combination had an insignificant increase in the flour strength, reduced bread volume, but had a high equal score of bread porosity in relation to L503.

In general, we can conclude that the Sr35+Sr25 combination had a lesser effect on the flour and bread indicators (except for the gluten content) (Table 6).

**Table 6.**
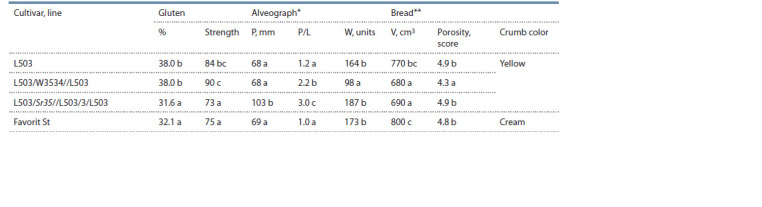
Bread making quality traits of spring bread wheat introgressive lines with the Sr22+Sr25 and Sr35+Sr25 genes combination and the cultivars L503 and Favorit (average for 2018–2020) * Indicators of the alveograph: P – dough tenacity, P/L – tenacity to extensibility ratio, W – flour strength; ** Indicators of bread evaluation: V – bread volume, porosity.

## Discussion

As noted above, all three studied genes Sr22, Sr25, Sr35 are effective against the biotypes of the P. graminis f. sp. tritici Ug99 race (http://rusttracker.cimmyt.org/?page_id=22). However, our studies have shown that only the combination of Sr22+Sr25 resistance genes is highly effective against the disease. There is reason to assume that in this case an additive effect or the so-called “forbidden combination” is manifested.

At the same time, in laboratory evaluation, the Sr35 gene is separately effective against the Saratov population of 2016, 2017 and 2020 and the Lysogorsk population of the Saratov region in 2018 and 2019 (O.A. Baranova, unpublished data), but the Sr35+Sr25 combination in the line L503/Sr35//L503/3/ L503 showed susceptibility to the stem rust causative agent. There can be several explanations for this phenomenon. Firstly, in laboratory studies, seedlings were infected, but under field conditions, during epiphytotiсs of the pathogen, adult plants were evaluated at the stages of the beginning of grain filling or milky-wax ripeness. It is possible that the Sr35 gene is resistant to this set of P. graminis f. sp. tritici populations only at the seedling stage. Secondly, it is possible that the Sr35 gene expression is suppressed by suppressor genes of cultivar L503. Similar cases were observed during the expression of the Sr21 gene also transferred from T. monococcum L. (The, Baker, 1975; on: Leonova, 2018).

Analyzing the influence of the Sr22+Sr25 and Sr35+Sr25 gene combinations on agronomic traits, primarily grain productivity and flour and bread quality, it is necessary to take into account well-known individual effects of the studied genes of resistance to the stem rust pathogen. The Sr25 gene was transferred to bread wheat as part of the 7DS-7DL-7Ae#1L translocation from chromosome 7Ae#1 of the tall wheatgrass, in which the following gene order was determined (from the centromere to the telomere end) – Sd1-Xpsr165-Xpsr105- αAmy-D2-Xpsr129-Lr19-Wsp-D1-Sr25-Y-Ep-D1 (Prins et al., 1996). Without a doubt, the entire translocation has an impact on agronomic performance.

Our early studies showed that this translocation is neutral in relation to grain yield, significantly increases the gluten content without changing its quality and does not affect dough tenacity, the tenacity to extensibility ratio of dough and flour strength. However, it significantly reduces the volume of bread with the same porosity. 7DS-7DL-7Ae#1L translocation did not affect the germination-earing period and plant height (Sibikeev et al., 2018). Thus, this translocation does not worsen agronomic performance.

It is known that the Sr22 gene was transferred into bread wheat from two diploid species carrying the A-genome – T. boeoticum Boiss. the source of G-21 (Gerechter-Amati et al., 1971) and T. monococcum the source of RL5244 (Kerber, Dyck, 1973). Transfers from these two sources include varying amounts of introgressed chromatin. The transfer from T. boeoticum contains almost entirely the long arm and part of the short arm of 7Аm (cv. Steinwedel), and from T. monococcum, the distal part of 7АmL (cv. Marquis) (Kerber, Dyck, 1973; Paull et al., 1994).

Due to the fact that the recombination between the A-genome of bread wheat and the Am-genomes of T. boeoticum and T. monococcum is limited due to the action of the Ph (pairing homeologous) gene system (Luo et al., 2000), introgressive material with the Sr22 gene is inherited as a single block in most cases. As shown by the studies of J.G. Paull et al. (1994), introgression with the Sr22 gene decreased grain yield and increased the germination-earing period. At the same time, the studies of T.T. The et al. (1988) revealed a slight decrease in grain productivity, depending on the recipient genotype (a decrease within 10 %). Successful attempts to reduce introgressive material with the Sr22 gene from T. boeoticum for the possible improvement of agronomic performance have been undertaken (Olson et al., 2010). From the available sources, it is not known about the study of the Sr22+Sr25 combination effect on agronomic performance and flour and bread quality. In addition, in our studies, we took a near isogenic line of the cultivar Marquis with the Sr22 gene from T. monococcum (W3534), which carries a smaller block of introgression material from 7Am.

In terms of grain yield, from three years of study, one year (2019) was a significant excess of the recipient cultivar L503, but one year (2020), there was a significant decrease, in general there were no differences, but a decrease in productivity was noted in absolute numbers (1154 kg/ha in introgression line with Sr22+Sr25 and 1422 kg/ha in cultivar L503). In terms of the germination-earing period, as in previous studies, there was an increase in the line with Sr22+Sr25 for four days, at the same time there was a decrease in the weight of 1000 grains, there were no differences in plant height.

In our studies, it was found that the line with Sr22+Sr25 has lower flour and bread making quality compared to the recipient cultivar L503, mainly due to the lower flour strength, coarser porosity and smaller volume of bread, at the same time, there was a high content of protein in grain – 16.9, against 16.4 % in L503.

Analyzing the Sr35+Sr25 combination, it should be noted that the Sr35 gene is localized on chromosome 3AL at 41.5 cm from the centromere (McIntosh et al., 1995) and has been studied in detail in terms of structure and regulation (Zhang et al., 2010; Saintenac et al., 2013), but, unfortunately, we could not find information about its effect on agronomic performance in the sources available to us. However, the absence of bread wheat commercial cultivars with this gene indicates a negative impact on the agronomic value (McIntosh et al., 2013).

In our studies, it was found that the line with the combination of Sr35+Sr25 genes in three years of study conceded in grain productivity only in 2020 and exceeded in 2018. In general, there were no significant differences; in absolute numbers, the grain productivity for the period 2018–2020 for the line with the combination Sr35+Sr25 was 1454 kg/ha, and for the cultivar L503 – 1422 kg/ha. In terms of the germination–earing period, the line with Sr35+Sr25 is earing two days later than the cultivar L503, a significant decrease in the weight of 1000 grains was noted, and there were no differences in plant height with the recipient cultivar L503. In terms of flour and bread making quality, the line with Sr35+Sr25 did not differ from the cultivar L503 except for a significant decrease in bread volume. In terms of protein content in grain, the line with Sr35+Sr25 did not significantly exceed L503 – 16.9 and 16.4 %, respectively.

## Conclusion

Thus, in general, for the entire studied complex of agronomic valuable traits, the combination of Sr35+Sr25 genes looks more effective than the line with the combination of Sr22+Sr25 genes. The study showed that the combination of Sr35+Sr25 genes does not worsen the agronomic performance of wheat; however, it is possible that the expression of the Sr35 gene in the L503/Sr35//L503/3/L503 line is suppressed by suppressor genes of the cultivar L503. It is necessary to further study the expression of the Sr35 gene in combination with other resistance genes, such as Sr31, and its use in Russian breeding programs.

## Conflict of interest

The authors declare no conflict of interest.
